# Optimization of a GMP-compliant automated one-pot synthesis of Al[^18^F]F-NOTA-Ubiquicidin_29 − 41_ for bacterial infection imaging by PET/CT

**DOI:** 10.1186/s41181-025-00370-7

**Published:** 2025-07-28

**Authors:** Anna Meyer, Thomas Ziegler, Jutta Moosbauer, Dirk Hellwig, Christian Fischer

**Affiliations:** Radiopharmacy, Department of Nuclear Medicine, University Hospital Regensburg, Regensburg, Germany

**Keywords:** ^18^F radiopharmaceuticals, Aluminum[^18^F]fluoride, Ubiquicidin, Antimicrobial peptides, Infection imaging

## Abstract

**Background:**

Bacterial infections and antimicrobial resistance constitute significant threats to global human health, resulting in millions of fatalities annually. The development of innovative diagnostic agents is essential to facilitate precision medicine approaches in the battle against infectious diseases. Infection imaging using radiolabeled antimicrobial peptides (AMP) has emerged as a promising approach to detect bacterial infections. UBI(29–41), an AMP fragment, exhibits binding to bacterial cell membranes. Conjugated to chelators, UBI(29–41) has been labeled with radiometals such as ^99m^Tc or ^68^Ga, and proven its ability to differentiate between sterile inflammation and infection with S. aureus by imaging. Due to its physical properties, ^18^F is more favorable for PET/CT imaging. As peptide labeling with ^18^F is challenging, we here implemented the Al^18^F labeling approach. This study aims to develop an optimized, fully automated, GMP-compliant process for radiolabeling of NOTA-conjugated UBI(29–41) with Al^18^F for PET/CT imaging of infections.

**Results:**

Optimized reaction conditions led to the establishment of a robust Al^18^Fcomplexation protocol, which was implemented on a SynChrom R&D module. The labeling reaction was carried out in an acidic medium (pH 4.0) at 105 °C for 15 min, followed by a two-step HPLC purification for 20 min. Optimization of reagent concentrations enabled an activity yield up to 10 ± 1 GBq, with a radiochemical yield of 24.2 ± 0.6% and an apparent molar activity of 45 ± 4 GBq/µmol at end of synthesis (EOS) (*n* = 3). The radiochemical purity was 96.6 ± 0.3% as determined by analytical HPLC, using UV absorption (220 nm). Quality control was successfully established using validated analytical procedures.

**Conclusions:**

The developed GMP-compliant radiolabelling process yields reproducible results with sufficient activities for further translation and investigations of clinical PET/CT imaging using Al[^18^F]F-NOTA-UBI(29–41) in infectious diseases.

## Background

Infectious diseases remain a significant global health challenge, largely due to the difficulties in localization and characterization of infectious foci. Although laboratory parameters such as white blood cell count, C-related peptide and procalcitonin provide guidance for clinical management, they do not offer information about the anatomical site of infection (Magrini et al. 2014). Conventional radiological imaging modalities, including ultrasound, Xray radiography, and computed tomography (CT), primarily detect morphological changes that are secondary to infection or associated inflammation. In contrast, nuclear medicine techniques such as positron emission tomography (PET) enable early diagnosis by visualizing physiological and biochemical alterations (Ferro-Flores et al. 2012).

^18^F FDG PET/CT is increasingly used for imaging of infection und inflammation, but it does not reliably distinguish between the two processes (Abikhzer et al. 2025). PET imaging utilizes radiopharmaceuticals, which consist of a biologically relevant carrier molecule labeled with a positron-emitting radionuclide such as fluorine-18 or gallium-68. However, the availability of infection-specific radiopharmaceuticals is limited, and most currently used agents are unable to reliably differentiate between infectious and sterile inflammatory processes.

**Fig. 1:**
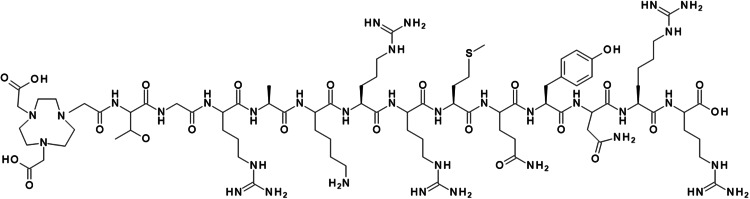
Chemical structure of NOTA-UBI_29-41_

Ubiquicidin (UBI), a cationic antimicrobial peptide fragment, has emerged as a promising agent for infection imaging due to its selective binding to anionic bacterial membranes (Ferro-Flores et al. 2016). In a mouse infection model, fulllength UBI_1 − 59_ injected intravenously demonstrated efficacy against methicillin-resistant S. aureus (MRSA), while a synthetic derivative composed of only eight amino acids (UBI_31 − 38_) showed somewhat reduced activity (Brouwer et al. 2006). Another UBI-derived peptide (UBI_29 − 41_) radiolabeled with technetium-99m, was used as a probe for real-time, whole-body scintigraphy to visualize infected tissues in mice (Brouwer et al. 2008). In an early clinical study involving seven adult patients with suspected bone or soft tissue infection, this method allowed for the identification of infection sites (Gandomkar et al. 2009). More recently, alternative radiolabeling strategies have been explored to enhance image quality, including conjugating UBI_29 − 41_ with the chelator 1,4,7-triazacyclononane-N, N’,N’’-triacetic acid (NOTA) at the N-terminus (Fig. 1). This precursor was complexed with gallium-68, a positron-emitting metallic radioisotope, in aqueous conditions (Santos et al. 2024; Mukherjee et al. 2018).

Compared to gallium-68, fluorine-18 offers several advantages for PET imaging, including lower positron energy (0.635 MeV), which results in shorter positron range and higher image resolution. Additionally, its longer half-life (109.8 min) permits centralized production and distribution, making it more suitable for routine clinical use. Cyclotron-based production also enables higher starting activity, further supporting its utility in clinical settings.

While fluorine-18 is traditionally introduced into molecules via covalent nucleophilic substitution, its non-metallic nature precludes direct complexation using conventional chelation strategies. The development of the aluminum[^18^F]fluoride (Al^18^F) labeling method by McBride et al. (2009) introduced a novel approach for incorporating fluorine-18 into stable complexes with thermodynamic and kinetic properties (Smith et al. 2011; Archibald and Allott 2021; Farkas et al. 2015). This reaction is highly pH-dependent, with optimal complexation occurring between pH 4 and 5, where aluminum exists as an octahedral hexahydrate. At higher pH values (pH > 5), a mixture of species with various ligands is formed, until the tetrahedral complex predominates beyond pH 6, insoluble, non-radioactive hydroxide complexes precipitate. Conversely, more acidic conditions (pH < 4) formation of [^18^F]HF is favored (Martin 1988).

**Fig. 2:**
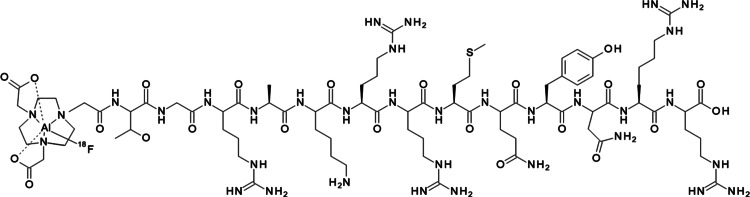
Chemical structure of Al[^18^F]F-NOTA-UBI_29 − 41_

For Al^18^F-labeling of NOTA-conjugated UBI_29 − 41_ (NOTA-Thr-Gly-Arg-Ala-Lys-Arg-Arg-Met-Gln-Tyr-Asn-Arg-Arg-OH), coordination occurs via three nitrogen atoms and two of the three carbonyl oxygen atoms, stabilizing the complex in serum for up to four hours at 37 °C (Fig. 2). However, competition between NOTA’s two acetate arms and Al[^18^F]F^2+^ for available coordination sites limits radiochemical yields to approximately 5–20% (D’Souza et al. 2011; André et al. 2002).

At the time this study was initiated (2022), data on the radiolabeling of NOTA-UBI_29 − 41_ with Al[^18^F]F^2+^ were scarce. A brief report by Ioppolo et al. (2018) mentioned the adaptation of a general Al[^18^F]F^2+^ peptide-labeling protocol for this tracer but did not provide any detailed information on reaction conditions, radiochemical yield, or product purity. The aim of the present study was to develop and optimize a GMP-compliant, fully automated synthesis method for Al[^18^F]FNOTA-UBI_29 − 41_ ensuring sufficient radiochemical yield and activity for clinical PET imaging applications.

## Methods

### Literature review

To establish optimal synthesis conditions for Al^18^Flabeled peptides, we reviewed existing methodologies for aluminum[^18^F]fluoride labeling published between 2018 and 2022 (Table 1). Key references included studies on the radiosynthesis of Al[^18^F]F-NOTA-Octreotide (Tshibangu et al. 2020; Dam et al. 2022) and Al[^18^F]F-PSMA-11 (Giglio et al. 2018; Kersemans et al. 2018; Lütje et al. 2019; Zha et al. 2021). Based on these studies, the labeling process can be divided into five main synthesis steps.

Purification of [^18^F]fluoride (step 1) was nearly identical across all protocols, typically involving an anion exchange cartridge. In contrast, the elution (step 2) employed a variety of eluents, including isotonic saline (0.9% NaCl), 0.5 M sodium acetate (NaOAc) buffer, or 0.05 M NaOAc buffer. The starting activities were in a wide range of 0.6–74 GBq. Formation of aluminum[^18^F]fluoride complex (step 3) was carried out in sodium acetate buffer (pH 4–5) at room temperature (21 °C) for 2–10 min. The radiolabeling step (step 4) was conducted under varying conditions: the incubation lasted 10–15 min, for NOTA-Octreotide, at 100–110 °C, whereas PSMA-11 labeling was performed at lower temperatures (up to 60 °C). The amounts of aluminum chloride and precursor used ranged from 20 to 210 nmol. Final purification was achieved using either high-performance liquid chromatography (HPLC) or solid phase extraction cartridges (SPE).

**Table 1:** Literature review

Ref.	Giglio et al. (2018)	Kersemans et al. (2018)	Lütje et al. (2019)	Tshibangu et al. (2020)	Zha et al. (2021)	Dam et al. (2022)
Complex	Al-[^18^F]F-PSMA11	Al-[^18^F]F-PSMA11	Al-[^18^F]F-PSMA11	Al-[^18^F]F-NOTA-Octreotide	Al-[^18^F]F-PSMA11	Al-[^18^F]F-NOTA-Octreotide
**Step 1**						
Fixing ^18^F	QMA Light	QMA Light	QMA Light	QMA Light	QMA Light	PS-HCO_3_
**Step 2**						
Elution ^18^F	0.5 M NaOAc (pH 4.5)	0.05 M NaOAc (pH 4.5)	0.05 M NaOAc (pH 4.5)	0.9% NaCl/ethanol (1:1, v/v)	0.9% NaCl	0.9% NaCl
**Step 3**						
^18^F activity	18.5–74 GBq	n.r.^a^	0.62–2.4 GBq	54 GBq	74–370 MBq	0.7-6 GBq
AlCl_3_	45 nmol	100 nmol	85 nmol	50 nmol	40 nmol	20 nmol
Reaction solution	0.5 M NaOAc buffer	0.05 M NaOAc buffer	0.5 M NaOAc buffer	0.1 M NaOAc buffer	0.05 M NaOAc buffer	0.5 M NaOAc buffer
pH	4.5	4.5	5	4.1	5	4.1
Temperature	21 °C	21 °C	n.r.	21 °C	n.r.	n.r.
Time	5 min	5 min	n.r.	2 min	n.r.	n.r.
**Step 4**						
Peptide	60 µg 63 nmol	200 µg 210 nmol	25 µg 24 nmol	120 µg 92 nmol	60 µg 46 nmol	26 µg 20 nmol
Temperature	50 °C	21 °C	50 °C	100 °C	60 °C	105 °C
Time	10 min	10 min	15 min	10 min	15 min	15 min
**Step 5**						
Purification	SPE	SPE	HPLC	SPE	SPE	SPE
Synthesis time	n.r.	35 min	n.r.	40 min	n.r.	n.r.
Radiochemical yield	18%	21 ± 3%	n.r.	26%	69.2 ± 5.4%	38 ± 8%
Molar activity	58–544 GBq/µmol	120 ± 28 GBq/µmol	n.r.	160.5 ± 75.3 GBq/µmol	n.r.	32 ± 10 GBq/µmol
Stability	4 h	4 h	n.r.	6 h	6 h	n.r.

^a^ n.r. indicates that this value was not reported in the literature

### Reagents and materials

All chemicals were commercially available. NOTA-UBI_29-41_ (1978.25 g/mol, net peptide) and UBI_29-41_ (1693.0 g/mol, net peptide) were purchased from ABX (Advanced Biomedical Compounds, Radeberg, Germany). Aluminum chloride hexahydrate, sodium acetate anhydrous, glacial acetic acid, and n-octanol were all of trace metal-free analytical grade. Absolute Ethanol (EtOH) met Ph. Eur. quality standards, and sterile, endotoxinfree Dulbecco’s phosphate-buffered saline was used for preparation. All listed chemicals were obtained from Merck (Darmstadt, Germany). Water for injection (WfI) and isotonic saline were pharmaceutical-grade products from B. Braun (Melsungen, Germany). Sep-Pak Accell Plus QMA Light cartridges were purchased from Waters (Eschborn, Germany).

Sodium acetate buffers at concentrations of 0.5 M and 0.05 M were prepared with varying pH values (3.5–6.5) by mixing the respective components in appropriate ratios to achieve the desired pH for manual elution testing. A 10 mM solution of AlCl_3_ was prepared in WfI, and aliquots were diluted in 0.5 mL of 0.5 M sodium acetate buffer adjusted to the required pH. The precursor NOTA-UBI_29-41_ was dissolved in water for injection and ethanol (EtOH) (90:10, v/v).

For preparative high-performance liquid chromatography, phosphate-buffered saline (PBS) was diluted 1:1 (v/v) with WfI to obtain a 6 mM phosphate buffer. The buffer was then mixed with ethanol at ratios of 98:2 and 90:10 (v/v), and the final pH was adjusted to 7.0 using a minimal volume of acetic acid.

Aqueous [^18^F]fluoride was produced via the ^18^O(p, n)^18^F nuclear reaction using an RDS 111 cyclotron (Siemens, USA). Enriched ^18^O-water (97%) for irradiation was obtained from AtaChem (Graz, Austria). A typical bombardment with a target current of 25 µA for 60 min yielded approximately 20 GBq of [^18^F]fluoride.

### Radiosynthesis

**Fig. 3:**
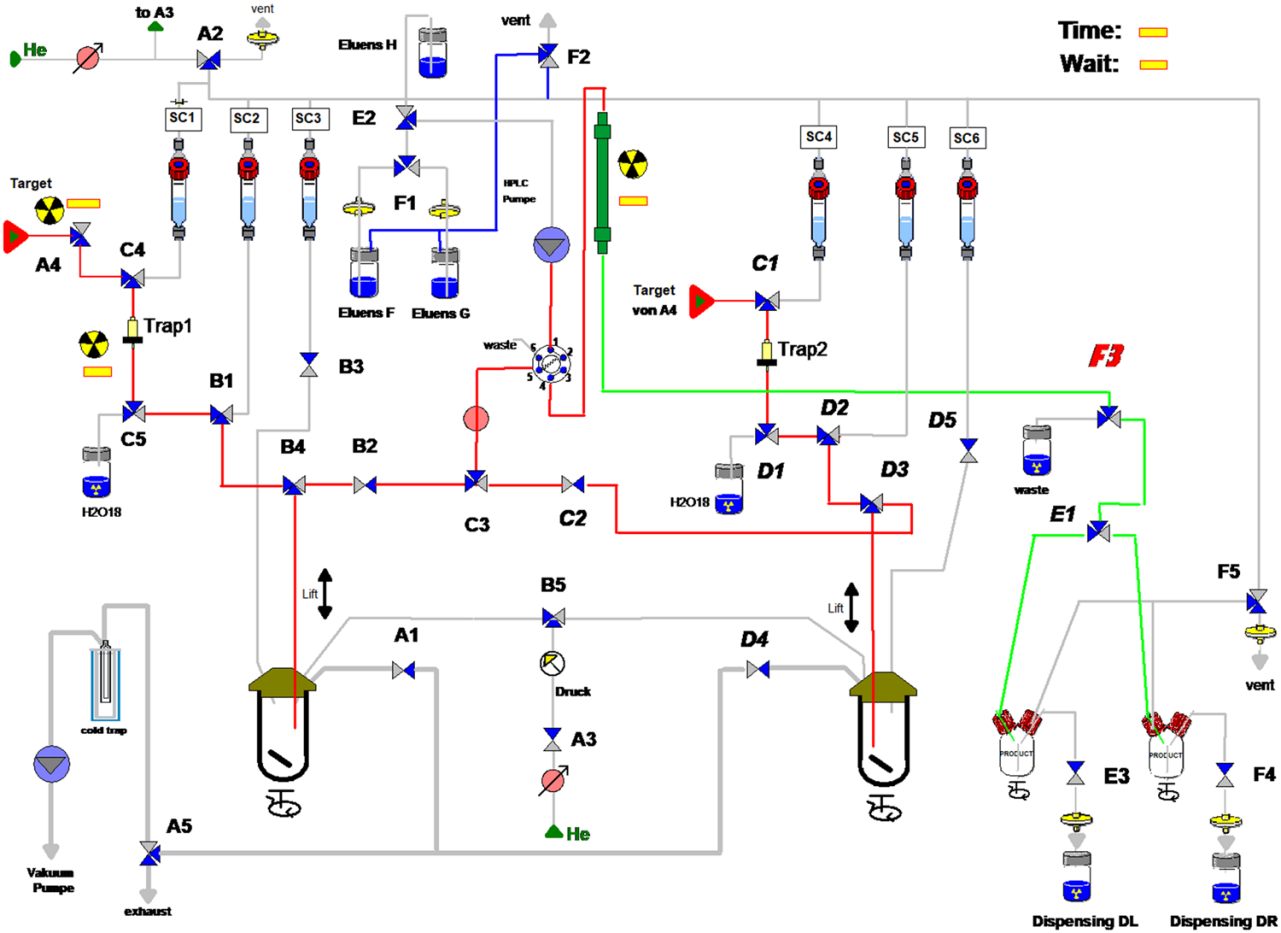
Schematics of the R&D synthesis module

The automated radiosynthesis of Al[^18^F]F-NOTA-UBI_29 − 41_ was performed as a two-step, one-pot reaction using a modified Elysia Raytest R&D Synchrom module (Straubenhardt, Germany). This module features a symmetrical dual-sided design, enabling the sequential execution of two syntheses per day (Fig. 3). Synthesis parameters such as temperatures and reaction times were programmed using the Gina Star software provided by the same manufacturer.

#### First step: [^18^F]fluoride separation

To obtain pure [^18^F]F^−^, the irradiated target water must first be processed. Each synthesis side of the module is equipped with an anion exchange cartridge (QMA Light) (Fig. 3, Trap 1 and 2) which selectively retains the [^18^F]F^−^ anions. The cartridge is subsequently rinsed with water via the target pathway to remove residual target material, and the used ^18^O-water is collected for recycling. Due to the time required for target delivery and trapping, the radiosynthesis is initiated 5 min after the end of bombardment.

#### Second step: [^18^F]fluoride elution

The purified [^18^F]fluoride is eluted into reactor 1/2 using 0.5 mL of optimal solution from SC 1/4, as determined by manual elution testing. The starting activity is immediately recorded by an integrated radioactivity detector.

#### Third step: aluminum[^18^F]fluoride formation

While stirring, a prediluted aluminum chloride (AlCl_3_) solution in 0.5 M NaoAc buffer (0.5 mL) is added from SC 2/5 to reactor 1/2. The aluminum[^18^F]fluoride complex forms at room temperature (21 °C) within 2 min under vacuum. To prevent side reactions, hydrogen [^18^F]fluoride, which may form at pH values below 4.0, is evaporated and removed during this step.

#### Fourth step: radiolabeling with NOTA-UBI_29 − 41_

After step 3, the reactor is vented, and the precursor solution from SC 3/6 is added. NOTA-UBI_29 − 41_ peptide complexes aluminum[^18^F]fluoride at 105 °C for 15 min. The presence of an organic co-solvent such as ethanol minimizes radiolysis and enhances radiochemical yield (D’Souza et al. 2011).

#### Fifth step: purification and formulation

After radiolabeling, the reaction mixture is cooled to 40 °C before subjected to purification by high-performance liquid chromatography. Product separation is manually triggered by activating valve F3, indicated by a real-time signal increase on the radioactivity detector located at the column outlet. The purified product is then diluted with 10 mL WfI in the corresponding collection vessel and transferred into the dispensing unit. The radiotracer is aseptically filled into sterile vials under type A cleanroom conditions with subsequent terminal sterile filtration followed by activity measurement. The HPLC column is finally regenerated using Eluent H, a mixture of acetonitrile and water (80:20, v/v), in accordance with the manufacturer’s specifications.

#### Preparative HPLC

Purification of Al[^18^F]F-NOTA-UBI_29 − 41_ was performed using a nonpolar VarioPrep Nucleodur C18 column (250 × 10 mm, Macherey-Nagel, Düren, Germany). Two sequential polar solvents with varying ethanol concentrations were applied at a flow rate of 5 mL/min over 20 min. During the first phase (0–10 min), phosphate buffer/ethanol (98:2 v/v, pH 7.0) was used to elute ionic impurities. In the second phase (10–20 min) phosphate buffer/ethanol (90:10 v/v, pH 7.0) facilitated the elution of the radiolabeled compound and other organic components (Table 2). Multiple syntheses were performed and analyzed using the preparative HPLC method to characterize and differentiate radioactive impurities.

**Table 2:** Conditions of HPLC for purification

Time (min)	Mobile phase	Flow (mL/min)
0–10	phosphate buffer 6 mM/ethanol (98:2), pH 7.0	5
10–20	phosphate buffer 6 mM/ethanol (90:10), pH 7.0	5

### Optimization of reaction parameters

#### General considerations

Initial reaction conditions (100 nmol AlCl_3_, pH 4.0, 500 µg precursor, 5% ethanol, 20 GBq [^18^F]fluoride, 15 min reaction time, and 105 °C reaction temperature; Table 5, column 2) were adapted from established aluminum[^18^F]fluoride labeling protocols for the peptides PSMA-11 and NOTA-Octreotide. Since this study focuses on labeling NOTA-UBI_29 − 41_, and because each peptide typically requires individual labeling conditions, a systematic optimization process was conducted. The parameters listed above were sequentially modified to identify the optimal synthesis conditions. The radiochemical yield (RCY) of Al[^18^F]F-NOTA-UBI_29 − 41_–amount of batch activity in relation to starting activity, decay-corrected to end of synthesis–served as the primary evaluation metric, with higher RCY indicating improved reaction efficiency. The combination of parameter yielding the highest RCY was selected for subsequent syntheses. Each synthesis, with the exception of the validation runs, was performed only once due to cost and time limitations. Quality control analyses were performed only during the validation phase of the final optimized process.

#### Manual elution tests

The optimal elution conditions for step 2 are defined by high elution capacity with minimal volume. To determine these conditions, freshly produced [^18^F]fluoride (~ 4 GBq in 1300 µL of enriched water) was diluted to 3 mL with ultrapure water. Aliquots (1 mL) of this solution were eluted through QMA light cartridges preconditioned with 10 mL of 0.5 M sodium acetate (pH 8.5) or 0.9% isotonic saline (pH 5.5). Considering that high concentrated solutions may compromise buffering capacity, additional tests were conducted using 0.05 M NaOAc. Furthermore, since sodium acetate solutions in previous studies typically had a pH of 4.5, elution efficiency was systematically evaluated across a range of pH values (4.0, 4.5, 5.0, 5.5, and 6.5) using 0.5 M NaOAc. Following trapping, the preconditioned QMA light cartridges were flushed with 10 mL WfI and subsequently dried prior to elution. Eluates were collected in 200 µL fractions, and cumulative radioactivity was quantified after each fraction using a dose calibrator.

#### Amount of aluminum chloride

To determine the optimal amount of aluminum chloride, nine syntheses were performed using 50, 100, 150, 175, 200, 225, 250, 300, and 350 nmol of aluminum chloride. The desired AlCl_3_ amount was achieved by diluting the required volume of the 10 mM AlCl_3_ stock solution with 0.5 mL 0.5 M acetate buffer.

#### Precursor concentration

NOTA-UBI_29 − 41_ was dissolved in 1 mL WfI/EtOH (90:10, v/v) to achieve final concentrations of 250, 400, 500, 600 and 750 µg/mL. Separate syntheses were carried out for each precursor concentration to assess its effect on labeling efficiency.

#### Starting activity of [^18^F]fluoride

To assess the impact of the activity on radiolabeling efficiency, five syntheses were conducted with intended target activities of 10, 20, 30, 40 and 50 GBq. Due to variations in cyclotron beam yield, the actual activities of [^18^F]fluoride were 13, 19, 29, 40 and 48 GBq, respectively.

#### pH optimization

As pH plays a critical role in the formation of aluminum[^18^F]fluoride complexes, syntheses were conducted at pH values of 3.5, 4.0, 4.5, 5.0 and 5.5 to determine the optimal pH range. The pH was adjusted using sodium acetate/acetic acid buffer solutions at the respective pH values.

#### Ethanol concentration

Ethanol concentrations of 0%, 2.5%, 5.0%, 7.5%, 10.0%, 12.5%, and 25.0% (v/v) were tested with respect to a total reaction volume of 2 mL. The ethanol content was adjusted by modifying the composition of the precursor solution (WfI/EtOH).

#### Reaction time and temperature

In step 4, NOTA-UBI_29 − 41_ complexes with aluminum[^18^F]fluoride at 105 °C in 15 min. To investigate alternative conditions, additional syntheses were carried out at 60 °C and 115 °C for 15 min. Furthermore, reaction times of 10 and 20 min were tested at 105 °C to assess the influence of incubation time on radiolabeling efficiency.

#### Preparative HPLC

In addition to the 90:10 (v/v) phosphate buffer/ethanol ratio, compositions of 88:12 and 92:8 (v/v) were also tested during the second purification step.

### Quality control

The appearance of the final product was assessed by visual inspection. pH was determined using pH-Fix indicator strips (pH-Fix 2.0–9.0, Macherey-Nagel, Düren, Germany) and compared against the manufacturer’s color reference scale. Aluminum content was determined by a limit test using QUANTOFIX^®^ test strips (Macherey-Nagel, Düren, Germany).

The identity of [^18^F]fluoride was confirmed by measuring its characteristic half-life (109.8 min ) using a dose calibrator (Isomed 2010, Nuvia, Dülmen, Germany). In addition, High Purity Germanium (HPGe) gamma spectrometry (Ametek, Pennsylvania, United States) was performed to verify the 511 keV photon peak. Radionuclide purity was assessed prior to product release and after complete decay to ensure the absence of long-lived radioactive contaminants. Endotoxin levels were measured using a portable Limulus Amoebocyte Lysate (LAL) test system (Endosafe^®^ nexgen-PTS™, Charles River, Massachusetts, United States) with preloaded quantification cartridges.

Sterility testing was performed by a contract laboratory in accordance with Ph. Eur. 2.6.1. guidelines. Ethanol, a potential residual solvent from the manufacturing process, and acetonitrile, possibly introduced from the preparative column regeneration solution acetonitrile/water (80:20), were quantified using gas chromatograph (GC 680, Agilent, Santa Clara, United States) equipped with a FS-OV-1701-CB-1 capillary column (50 m x 0.4 mm, CS-Chromatography Service GmbH, Langerwehe, Germany).

Chemical and radiochemical purity (RCP), as well as compound identity, were assessed using an Agilent 1200 HPLC system (Santa Clara, United States) equipped with a diodenarray detector (220 nm) and a shielded sodium iodide (NaI) scintillation detector. Chromatographic separation was achieved on an Onyx Monolithic C18 column (100 × 4.6 mm, Phenomenex, Torrance, United States) at 30 °C with a flow rate of 1 mL/min. The mobile phase consisted of water with 0.1% trifluoroacetic acid (A) and acetonitrile (Merck KGaA, Darmstadt, Germany) (B). A sample volume of 20 µL was injected. The elution gradient is detailed in Table 3.

**Table 3:** Elution gradient analytical HPLC method

Time [min]	A: Water + 0.1% TFA [%]	B: Acetonitrile [%]
0–3	98	2
3–18	98 → 85	2 → 15
18–20	85	15

The NOTA-conjugated peptide (NOTA-UBI_29 − 41_) and the unmodified peptide fragment (UBI_29 − 41_), each prepared at 250 µg/mL in formulation solution, served as reference standards. The HPLC method was validated for specificity, linearity, precision, and for the limits of detection (LOD) and quantification (LOQ). For UBI_29 − 41_, the LOD and LOQ were determined to be 0.986 µg/mL and 2.938 µg/mL, respectively, for NOTA-UBI_29 − 41_, the values were 1.044 µg/mL and 3.107 µg/mL.

Since the reference standard AlF-NOTA-UBI_29 − 41_ is not commercially available, its LOD and LOQ could not be determined. To nevertheless confirm the identity of the radioactive Al[^18^F]F-NOTA-UBI_29 − 41_, the corresponding non-radioactive analogue [^19^F]AlF-NOTA-UBI_29 − 41_ was synthesized using the same production process, substituting [^18^F]fluoride with potassium fluoride.

To verify that [^18^F]fluoride is not retained on the column during analysis, a recovery experiment was performed by spiking a sample of Al[^18^F]F-NOTA-UBI_29 − 41_ with 5% free [^18^F]fluoride. Subsequent analysis confirmed a recovery of 5%, demonstrating that [^18^F]fluoride elutes quantitatively under the applied chromatographic conditions.

The acceptance criteria for the final radiopharmaceutical depicted in Table 4 were defined in accordance with the Ph. Eur. monographs for other gallium-68 labeled radiopharmaceuticals and aluminum-containing medicinal products.

**Table 4:** Test parameters, acceptance criteria, and analytical methods for Al[^18^F]F- NOTA-UBI_29 − 41_

Parameter	Method	Acceptance criteria
Appearance	Visual inspection	clear, colorless
pH	pH strip	4.5–8.5
Batch radioactivity	Dose calibrator	10–1500 MBq/mL
Half-life [^18^F]	Dose calibrator	1.83 h ± 4.54%
Radionuclide purity	Gamma spectrum	511 keV **≥** 99,9%
Al[^18^F]F-NOTA-UBIquicidin_29-41_	HPLC NaI	≥ 95.0%
∑ ^18^F-impurities		≤ 5.0%
NOTA-Ubiquicidin_29-41_	HPLC UV	≤ 50 µg/mL
Ubiquicidin_29-41_		≤ 50 µg/mL
Ethanol	GC	≤ 10.0%
Acetonitrile		≤ 410 µg/mL
Aluminum	Quantofix test strip	≤ 5 µg/mL
Sterility	Ph. Eur. 2.6.1	sterile
Bacterial Endotoxins	LAL-Test	< 17.5 E.U./mL

#### Process validation

The final synthesis procedure, including all reagent quantities, was validated through three consecutive production runs under identical conditions to confirm reproducibility and suitability for routine clinical application. Each batch was tested according to the specifications listed in Table 4.

#### Stability in final formulation

To evaluate the stability of Al[^18^F]FNOTA-UBI_29 − 41_ in its final formulation, the radiopharmaceutical was stored at room temperature for 13 h. Quality control parameters were assessed every two hours over the initial 6 h period, corresponding approximately to the half-life. Thereafter, RCP was monitored by HPLC at 2-hour intervals for an additional 7 h.

#### Distribution coefficient

The distribution coefficient (LogD_7.4_) of Al[^18^F]F-NOTA-UBI_29 − 41_ was determined using a standard n-octanol/PBS partitioning assay. A 100 µL aliquot of the radiolabeled complex (0.5 MBq) was mixed with 400 µL PBS (pH 7.4) and 500 µL n-octanol. The biphasic mixture was vortexed for 2 min and centrifuged at 3000 rpm for 5 min to facilitate phase separation. Subsequently, 100 µL of the organic phase and 10 µL of the aqueous phase were collected. Radioactivity in each sample was measured over 300 s using a HPGe gamma detector and analyzed with Gamma Vision LVis software (Ametek, Pennsylvania, United States). Measured counts were decay-corrected and normalized for volume and measurement duration prior to calculation. The distribution coefficient was calculated according the following equation: $\:{LogD}_{7.4}=log\frac{cps\:\left(organic\:phase\right)}{cps\:\left(aqueous\:phase\right)}$

## Results

The initial automated radiosynthesis of Al[^18^F]F-NOTA-UBI_29 − 41_ under starting conditions yielded a radiochemical yield of 8% at end of synthesis (Table 5, column 2). In subsequent experiments, the condition that achieved the highest RCY in each series was retained for further optimization. The optimized reaction parameters are presented in Table 5, column 3. 

**Table 5:** Initial vs. optimized reaction parameters

Parameters	Initial	Optimized
Amount of AlCl_3_ [nmol]	100	175
Precursor concentration [µg/mL]	500	500
Starting activity of [^18^F]fluoride [GBq]	20	19
pH	4.0	4.0
Ethanol concentration [%]	5	5
Reaction time [min]	15	15
Reaction temperature [°C]	105	105
Activity yield (EOS) [GBq]	1.6	3.8
Radiochemical yield (EOS) [%]	8	20

### Manual elution tests

**Fig. 4:**
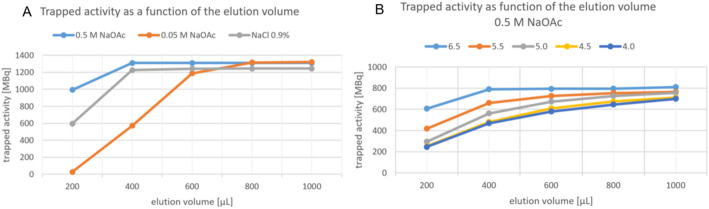
Elution patterns of: **A**: [^18^F]fluoride from QMA light cartridge using 0.5 M NaOAc, 0.05 M NaOAc, and 0.9% NaCl. **B**: [^18^F]fluoride from QMA light cartridge using 0.5 M NaOAc at various pH values

Elution efficiency of [^18^F]fluoride from QMA light cartridges was evaluated using various eluents. Both 0.5 M sodium acetate and 0.9% NaCl quantitatively eluted the radioactivity with 400 µL. However, 0.05 M NaOAc required a larger volume (800 µL). While 0.9% NaCl was effective, its elution efficiency was approximately 4% lower than that of 0.5 M NaOAc (Fig. 4A). Elution performance of 0.5 M NaOAc was highest at native pH (8.5) but decreased with acidification (Fig. 4B).

### Amount of aluminum chloride

**Fig. 5:**
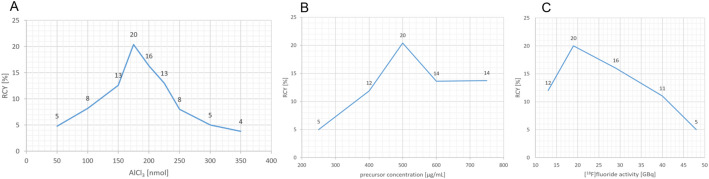
Radiochemical yield (RCY) of Al[^18^F]F-NOTA-UBI_29 − 41_ as a function of: **A**: the amount of AlCl_3_, **B**: the precursor concentration and **C**: the [^18^F]fluoride activity

The radiochemical yield of Al[^18^F]F-NOTA-UBI_29 − 41_ increased with rising amounts of aluminum chloride, reaching a maximum of 20% at 175 nmol (Fig. 5A). Beyond this point, further increases in AlCl_3_ resulted in a decline in yield. Therefore, 17.5 µL of a 10 mM aluminum chloride solution was selected for subsequent syntheses.

### Precursor concentration

RCY improved with increasing precursor concentrations up to 500 µg/mL, where a maximum yield of 20% was observed. Further increases led to a gradual decline (e.g., 14% at 600 µg/mL), with no improvement beyond (Fig. 5B).

### Starting activity of [^18^F]fluoride

The highest radiochemical yield was achieved with a starting activity of 19 GBq [^18^F]. Activities above this level led to decreased yields. (Fig. 5C)

### pH optimization

**Fig. 6:**
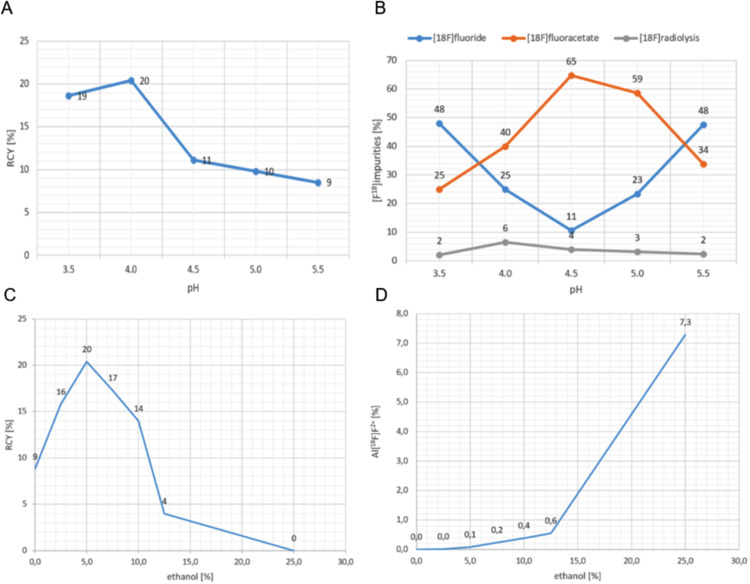
Influence of reaction conditions on the radiochemistry of Al[^18^F]F-NOTA-UBI_29 − 41_. **A**: Radiochemical yield (RCY) as function of pH, **B**: Distribution of co-products at different pH values, **C**: RCY as a function of ethanol concentration, **D**: Proportion of the Al[^18^F]F^2+^ species as a function of ethanol concentration

The maximum radiochemical yield (20%) of Al[^18^F]F-NOTA-UBI_29 − 41_ was achieved at pH 4.0 (Fig. 6A). At this pH, a distinct increase in radiolysis-associated byproducts (6%) was also observed (Fig. 6B, grey line). The profiles of [^18^F]fluoride (Fig. 6B, blue line) and [^18^F]fluoroacetate (Fig. 6B, orange line) exhibit an inverse relationship in response to pH changes. The equilibrium between these two species was markedly pH-dependent, with pH 4.5 promoting [^18^F]fluoroacetate (65%) formation while concurrently reducing the level of unbound [^18^F]fluoride (11%).

### Ethanol concentration

The addition of 5% ethanol (100 µL), increased the RCY of Al[^18^F]F-NOTA-UBI_29 − 41_ from 9% (no ethanol) to 20%. Concentrations above 5% progressively reduced the yield; at 25%, labeling failed entirely (Fig. 6C). Concurrently, a higher ethanol content promoted formation of Al[^18^F]F^2+^ species (Fig. 6D).

### Reaction time and temperature

A reaction time of 15 min at 105 °C produced optimal results. Shorter durations (e.g. 10 min) resulted in insufficient labeling (2% RCY), while extending to 20 min slightly decreased RCY (16%). A reaction temperature of 60 °C yielded no product, and at 115 °C, yielded dropped by half (10% RCY).

### Preparative HPLC

**Fig. 7:**
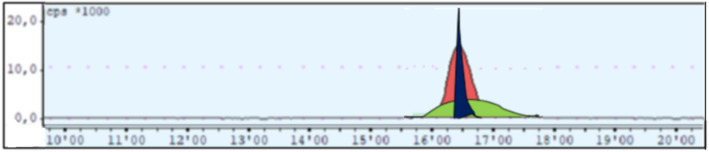
Preparative HPLC radiochromatogram of the second purification step with different phosphate buffer-ethanol-ratios (v/v): 92:8 (green), 88:12 (blue) and 90:10 (red)

The optimal phosphate buffer/ethanol ratio for the second purification step was determined to be 90:10 (v/v). A composition of 92:8 (v/v) resulted in broadened peaks and prolonged elution (~ 2 min), ultimately increasing the final batch volume to 20 mL – the maximum capacity of the collection vial (Fig. 7, green). Conversely, a ratio of 88:12 (v/v) led to small peaks and failed to achieve adequate separation between radiolabeled product and co-eluting organic impurities (Fig. 7, blue). The 90:10 (v/v) mixture offered the best compromise between peak resolution and collection volume, enabling efficient isolation of Al[^18^F]F-NOTA-UBI_29 − 41_ (Fig. 7, red).

**Fig. 8:**
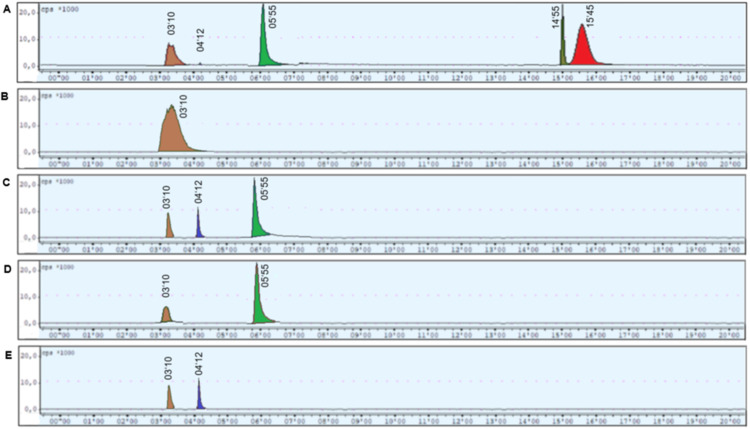
Preparative HPLC radiochromatograms of: **A.** Al[^18^F]F-NOTA-UBI_29 − 41_, **B.** [^18^F]fluoride, **C.** synthesis without precursor, **D.** synthesis without AlCl_3_ and precursor, **E**. synthesis using phosphate citrate buffer

Al[^18^F]F-NOTA-UBI_29 − 41_ exhibited a retention time of 16.45 min in the preparative HPLC radiochromatogram. A secondary peak at 14.55 min, eluting approximately one minute earlier, is presumed to correspond to a radiolysis byproduct of the peptide (Fig. 8A). Free [^18^F]fluoride was identified at 3.10 min, as confirmed by comparison with [^18^F]fluoride obtained directly from the cyclotron target (Fig. 8B). A distinct peak at 4.12 min was attributed to unbound aluminum[^18^F]fluoride, as evidenced by increasing signal intensity in a control synthesis performed in the absence of the precursor (Fig. 8C).

An additional impurity was observed at 5.55 min. To elucidate its origin, a control synthesis was conducted in the absence of both aluminum chloride and precursor, using only [^18^F]fluoride and 0.5 M sodium acetate buffer. Under these conditions, the 5.55 min peak remained detectable (Fig. 8D), suggesting a potential association with acetate-mediated processes. To further investigate this, sodium acetate was replaced with a phosphate-citrate buffer of equivalent pH. In the resulting radiochromatogram, only peaks corresponding to free [^18^F]fluoride (3.10 min) and unbound aluminum-[^18^F]fluoride (4.12 min) were observed, while the 5.55 min peak was no longer present (Fig. 8E), confirming the acetate-dependency of this radiochemical impurity.

### Upscaling of final batch activity

During process optimization, an inverse correlation was observed between the starting activity of [^18^F]fluoride and the radiochemical yield under constant reaction conditions. Doubling the initial [^18^F]fluoride activity from 19 GBq to 40 GBq resulted in a comparable activity yield of approximately 4 GBq, while the RCY significantly decreased from 20 to 11%. To counteract this decline, the contents of key reaction components, including aluminum chloride and NOTA-UBI_29 − 41_, were adjusted proportionally to the starting activity (Table 6). The optimized reaction parameters–pH 4.0, 5% ethanol, 105 °C, and a reaction time of 15 min–were maintained throughout all experiments. Despite these modifications, RCY could not be improved beyond 20 ± 1%. Nevertheless, by fine-tuning the precursor concentration, the activity yield was successfully increased to 8 GBq without compromising the radiochemical yield.

**Table 6:** Optimized vs. upscaled reaction parameters

Parameters	Optimized	Upscaled
Amount of AlCl_3_ [nmol]	175	350
Precursor concentration [µg/mL]	500	1000
Starting activity of [^18^F]fluoride [GBq]	19	38
pH	4.0	4.0
Ethanol concentration [%]	5	5
Reaction time [min]	15	15
Reaction temperature [°C]	105	105
Activity yield (EOS) [GBq]	3.8	8.1
Radiochemical yield (EOS) [%]	20	21

### Quality control

**Fig. 9:**
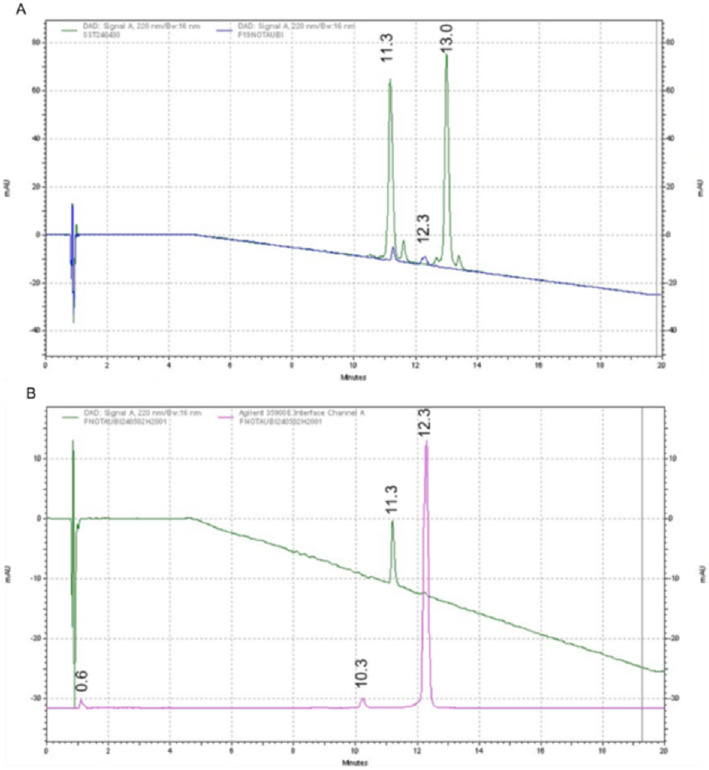
Representative chromatograms of the reference compounds (**A**) and the radiolabeled product Al[^18^F]F-NOTA-UBI_29 − 41_ (**B**). **A**: UV chromatogram of Al[^19^F]F-NOTA-UBI_29 − 41_ (12.3 min, blue), UBI_29 − 41_ (11.3 min, green) and NOTA-UBI_29 − 41_ (13.0 min, green) detected with UV **B**: Radiochromatogram of Al[^18^F]F-NOTA-UBI_29 − 41_ (12.3 min, pink) along with radioactive impurities (0.6 min and 10.3 min, pink) detected with NaI, and the unmodified peptide fragment UBI_29 − 41_ (11.3 min, green) detected with UV

Analytical UV chromatograms of the reference compounds show the retention times of the NOTA-conjugated precursor (NOTA-UBI_29 − 41_, RT 13.0 min) and the unmodified peptide fragment (UBI_29 − 41_, RT 11.3 min), both detected in the green line. The non-radioactive analog Al[^19^F]F-NOTA-UBI_29 − 41_ exhibited a retention time of 12.3 min (blue line) (Fig. 9A). Figure 9B presents the analytical HPLC chromatogram of Al[^18^F]F-NOTA-UBI_29 − 41_. The radioactive product (Al[^18^F]F-NOTA-UBI_29 − 41_, 12.3 min) and two additional radioactive impurities (RT 0.6 min and 10.3 min) were detected using NaI scintillation detection (pink line). The non-radioactive peptide UBI_29 − 41_ was identified at 11.3 min via UV detection (green line). Compound identities were confirmed by coelution with the corresponding non-radioactive reference standards NOTA-UBI_29 − 41_ and UBI_29 − 41_ (Fig. 9A).

### Validation

Validation runs were performed using the upscaled synthesis protocol, and the corresponding batch results are summarized in Table 7. Al[^18^F]F-NOTA-UBI_29 − 41_ was produced with a final batch activity of 10 ± 1 GBq and an average radiochemical yield of 24.2 ± 0.6% (EOS) within 45 min.

All three validation batches (A-C) met the predefined product specifications achieving a radiochemical purity of 96.6 ± 0.3%. The apparent molar activity (A_m_) was found to be 45 ± 4 GBq/µmol. As Al[^19^F]F-NOTA-UBI_29 − 41_ was not available as a reference standard and could not be validated for use with the analytical HPLC method, A_m_ was calculated based on the total amount of NOTA-UBI_29 − 41_ and UBI_29 − 41_. Since NOTA-UBI_29 − 41_ was not detected in any of the three batches, the LOQ (3,107 µg/mL) was used for calculation.

The pH of the final product was consistently 7.0. The ethanol content was 3.1 ± 0.3%, and residual acetonitrile was present at concentration of 13.8 ± 2 µg/mL. Aluminum content was consistently ≤ 5.0 µg/mL. The radionuclide identity was confirmed using gamma spectrometry and with half-life measurement using a dose calibrator. All three batches were sterile and complied with bacterial endotoxins limits.

**Table 7:** Validation runs batch analysis

Parameters	Acceptance criteria	Batch A	Batch B	Batch C
Starting activity [^18^F]fluoride [GBq]		36	43	43
Activity yield (EOS) [GBq]		9	11	10
Radiochemical yield (EOS) [%]		24.0	24.8	23.8
Batch volume [mL]		15.8	17.1	14.5
Appearance	Clear, colourless	conforms	conforms	conforms
pH	4.5–8.5	7.0	7.0	7.0
Half-life [h]	1.83 ± 4.54%	1.84	1.83	1.83
Radionuclidic purity [%]	≥ 99.9	conforms	conforms	conforms
Al[^18^F]F-NOTA-UBI_29 − 41_ [%]	≥ 95.0	96.4	96.5	96.9
[^18^F]Impurities [%]	≤ 5.0	3.6	3.5	3.1
NOTA-UBIquicidin_29 − 41_ [µg/mL]	≤ 50	< LOQ	< LOQ	< LOQ
Ubiquicidin_29 − 41_ [µg/mL]	≤ 50	18.5	23.8	20.8
Ethanol [%]	≤ 10.0	2.8	3.4	3.1
Acetonitrile [µg/mL]	≤ 410	11.8	15.0	14.5
Aluminum[µg/mL]	≤ 5.0	conforms	conforms	conforms
Sterility	sterile	conforms	conforms	conforms
Bacterial Endotoxines [E.U./mL]	< 17.5	< 1.0	< 1.0	< 1.0

#### Stability in final formulation

**Fig. 10:**
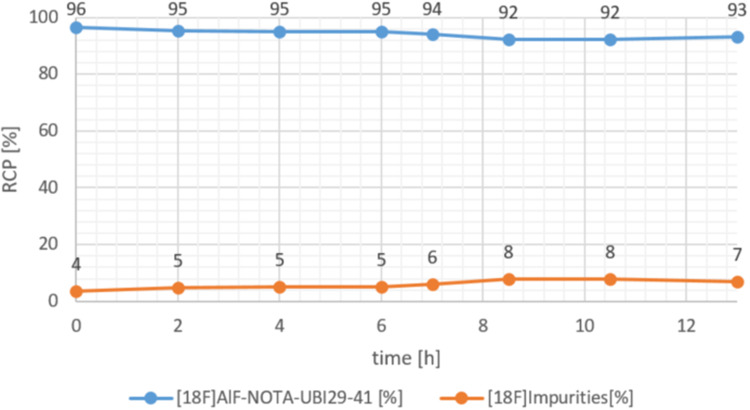
Stability of Al[^18^F]F-NOTA-UBI_29 − 41_ (blue) and [^18^F]impurities (orange) over a period of 13 h

The stability of Al[^18^F]F-NOTA-UBI_29 − 41_ in the formulation solution was determined using the validated analytical HPLC method. It was demonstrated that radiochemical purity remained at 95% for up to 6 h post-synthesis, with all quality parameters remaining within the specified limits. However, after 13 h, RCP declined to 93% (Fig. 10).

#### Distribution coefficient

The distribution coefficient (LogD_7.4_) of Al[^18^F]F-NOTA-UBI_29 − 41_ was determined to be -2.9 ± 0.1 (*n* = 3), indicating high hydrophilicity and suggesting predominant renal clearance pathway.

## Discussion

Optimization of the radiolabeling process identified several critical factors influencing radiochemical yield.

The efficacy of [^18^F]fluoride elution from the QMA Light cartridge was found to be pH-dependent, as demonstrated by manual experiments using various eluents. Lower pH values increased oxonium ion concentrations, resulting in higher acidity and reduced availability of counter-ions necessary for efficient [^18^F]fluoride elution (Fig. 4B). This pH sensitivity has not been previously reported by Giglio et al. (2018), Kersemans et al. (2018) or Lütje et al. (2019), all of whom successfully employed 0.5 M sodium acetate at pH 4.5 without observing elution inefficiencies. Due to this observed pH dependency and the potential of concentrated acetate buffers (e.g. 0.5 M, pH 8.5) to alter the reaction pH, isotonic saline was selected as the eluent of choice.

According to Martin (1988), pH values below 4.0 favor [^18^F]HF formation, while elevated pH values can promote the precipitation of non-radioactive aluminum hydroxide species. This precipitation limits the availability of Al^3+^ ions, impairs complex formation with aluminum-[^18^F]fluoride, and consequently reduces the radiochemical yield of Al[^18^F]F-NOTA-UBI_29 − 41_.

The addition of small amounts of ethanol–a biocompatible radiolysis inhibitor–improved radiochemical yield, consistent with the findings of D’Souza et al. (2011). However, increasing the ethanol concentrations beyond 5% negatively impacted RCY, likely due to its denaturing effects on the peptide’s tertiary structure, which impaired complexation and increased the proportion of unbound aluminum[^18^F]fluoride (Fig. 6C). A final ethanol concentration of 5% was identified as optimal.

Efficient complexation of Al[^18^F]F^2+^ with NOTA-conjugated peptides required a reaction temperature of 100–110 °C and a reaction time of approximately 15 min. At lower temperatures (e.g., 60 °C, used for PSMA-11 labeling), no complexation was observed. Similarly, deviations from the 15-minute reaction time significantly reduced radiochemical yield, underscoring the specificity of the chelation to these parameters.

Upscaling experiments revealed a delicate balance between the key reactants (AlCl_3_, NOTA-UBI_29 − 41_ and [^18^F]fluoride). Both the ^18^F−-to-Al^3+^ and Al^3+^-to-precursor ratios were found to be critical for efficient complexation (Cleeren et al. 2016). In line with Laverman et al. (2012), reduced amounts of Al^3+^ impaired the formation of the aluminum-[^18^F]fluoride complex and led to higher levels of unbound [^18^F]fluoride. Conversely, excessive of AlCl_3_ promoted formation of non-radioactive aluminum species, reducing radiochemical yield. When using 19 GBq [^18^F]fluoride and an AlCl_3_/peptide ratio of 0.7 (175 nmol/253 nmol), we obtained 3.8 GBq Al[^18^F]F-NOTA-UBI_29 − 41_ with a radiochemical yield of 20%, decay-corrected to the end of synthesis. Increasing the precursor concentration above 500 µg/mL (253 nmol) did not further improve yield, suggesting system saturation. Similarly, higher [^18^F]fluoride activity did not enhance labeling efficiency and instead shifted the equilibrium toward the formation of alternative aluminum fluoride species (e.g. Al[^18^F]F^+^, Al[^18^F]F_3_) that are less efficiently chelated by NOTA-UBI_29 − 41_ (Baker 1968).

No further improvements in RCY were achieved beyond the optimized conditions (pH 4.0, 5% ethanol, 105 °C, 15 min). However, by doubling the concentrations of key reagents (350 nmol AlCl₃, 1 mg precursor, 38 GBq [^18^F]fluoride), the activity yield increased by ~ 50%, from 3.8 GBq to 8.1 GBq. These upscaled synthesis process was validated in three independent identical GMP-compliant production runs, consistently yielding 10 ± 1 GBq with a radiochemical yield of 24.2 ± 0.6% (EOS). This result is in line with previously reported Al^18^F labeling efficiencies and reflects the intrinsic coordination competition within the NOTA chelator (D’Souza et al., 2011).

While SPE methods can yield higher activity, preparative HPLC offers superior separation of the target compound from chemical and radiochemical impurities, including unlabeled AlF-NOTA-UBI_29 − 41_. Although approach results in a lower batch yield, it ensures higher radiochemical purity. The presence of unlabeled AlF-NOTA-UBI_29 − 41_ may compromise binding specificity by occupying saturable targets, potentially leading to an underestimation of receptor density (low A_m_). Conversely, excessively high A_m_ can result in substantial tracer uptake in non-target issues via high-affinity, low-capacity binding sites, particularly during first-pass distribution (Vermeulen et al. 2019). In our synthesis, the apparent molar activity was 45 ± 4 GBq/µmol, which lies within the reported range for other Al^18^F-labeled tracer (27–160 GBq/µmol, Table 1). Further studies are warranted to evaluate the impact of this moderate molar activity on in-vivo targeting performance.

The identity of Al[^18^F]F-NOTA-UBI_29 − 41_ in the preparative HPLC (RT 16.45 min) was confirmed by co-elution of the non-radioactive analogue Al[^19^F]F-NOTA-UBI_29 − 41_ using analytical HPLC. Peaks corresponding to [^18^F]fluoride (3.10 min) and unbound aluminum[^18^F]fluoride (4.12 min) were confirmed via control experiments. A previously reported peak at 5.55 min–assigned to aluminum[^18^F]fluoride by Giglio et al. (2018)–was also observed. However, this peak remained present even in the absence of aluminum chloride, contradicting that assignment. Its persistence in reactions containing only [^18^F]fluoride and 0.5 M sodium acetate buffer suggested the formation of [^18^F]fluoroacetate. Replacing sodium acetate with a phosphate-citrate buffer eliminated this peak, confirming acetate’s essential role in its formation. However, phosphate-citrate buffer was found unsuitable for Al[^18^F]F-NOTA-UBI_29 − 41_ complexation, as citrate strongly binds aluminum, inhibiting complexation (Rajan et al. 1981).

As expected, Al[^18^F]F-NOTA-UBI_29 − 41_ exhibited good formulation stability, maintaining > 95% radiochemical purity for at least six hours post-synthesis. This is attributable to the robust aluminum[^18^F]fluoride-NOTA complex and is consistent with the stability reported for Al[^18^F]F-NOTA-Octreotide (> 96% after six hours; Tshibangu et al. 2020). In contrast, peptides labeled with aluminum[^18^F]fluoride lacking a NOTA chelator, such as Al[^18^F]F-PSMA-11, demonstrate lower stability (90–95% after 4 h; Kersemans et al. 2018; Giglio et al. 2018). Use beyond the 6-hour timeframe is not anticipated, as clinical application is restricted to the standard on-site patient care schedule. Consequently, no additional studies have been conducted to evaluate stability beyond this period.

At early 2025, and thus following the development and implementation of our synthesis process (2022–2024) based on the literature review summarized in Table 1, Jiang et al. (2025) reported the synthesis of a novel Al[^18^F]-labeled, NOTA-modified UBI_29 − 41_ derivative, in which the NOTA chelator was conjugated to the amino group of a lysine side chain rather than to the N-terminus of the peptide. They used significantly larger amounts of aluminum and precursor while employing considerably lower starting activities (130 ± 59.2 MBq vs. 40 GBq). Radiolabeling was performed in a potassium hydrogen phthalate buffer (pH 4) for 1 h at 90 °C. Despite these conditions, they achieved a RCY of 24.3 ± 6.9%, comparable to our result. Purification was performed using both SPE (C18 cartridge) and preparative HPLC (RCP of 99.0 ± 0.8%) whereas we employed only preparative HPLC (96.6 ± 0.6%). While Jiang et al. focused on preclinical evaluation with lower activities and metabolic analysis, we established a GMP-compliant, fully automated production process with high activity yields suitable for clinical use.

## Conclusion

In this study, a robust and GMP-compliant automated synthesis of Al[^18^F]F-NOTA-UBI_29 − 41_ was successfully established. The aluminum[^18^F]fluoride labeling approach combines the advantages of chelator-based radiolabeling with the favorable imaging characteristics of [^18^F]fluoride. Radiolabeling of the NOTA-conjugated antimicrobial peptide UBI_29 − 41_ with [^18^F]fluoride represents a promising strategy for PET/CT imaging of bacterial infections.

The final automated synthesis yielded Al[^18^F]F-NOTA-UBI_29 − 41_ with a radiochemical yield of 24.2 ± 0.6% (EOS) and a radiochemical purity of 96.6 ± 0.3% within 45 min, providing sufficient activity for clinical use. The process has been fully validated, quality control methods have been implemented, and formulation stability is confirmed. The established GMP-compliant radiolabeling protocol provides a foundation for further studies and potential clinical translation of this novel infection-specific PET tracer.

## Data Availability

The datasets used and/or analyzed during the current study are available from the corresponding author on reasonable request.

## Abbreviations

A_m_: Molar activity
AlCl_3_: Aluminum chloride
Al^18^F: Aluminum [^18^F]fluoride
AMP: Antimicrobial peptides
CT: Computed tomography
DAD: Diodenarray detector
EtOH: Ethanol absolute
EOS: End of Synthesis
GC: Gas chromatography
HPGe: High Purity Germanium Radiation Detector
HPLC: High-performance liquid chromatography
LAL: Limulus Amoebocyte Lysate
LOD: Limit of detection
LogD: Distribution coefficient
LOQ: Limit of quantification
min: Minute
MRSA: Methicillinresistant S. aureus
0.9% NaCl: Isotonic saline
NaOAc: Sodium acetate
NaI: Sodium iodide
NOTA: 1,4,7triazacyclononaneN, N’,N’’triacetic acid
PBS: Phosphate buffered saline
PET: Positron emission tomography
RCP: Radiochemical purity
RCY: Radiochemical yield
SPE: Solid phase extraction
SST: System Suitability Test
UBI: Ubiquicidin
WfI: Water for injection
